# An efficient Pulsed-Field Gel Electrophoresis (PFGE) method for typing autolytic *Lacticaseibacillus rhamnosus* strains

**DOI:** 10.1016/j.mex.2022.101945

**Published:** 2022-11-29

**Authors:** Jouni Heikkinen, Atte von Wright, Ivana Nikodinoska, Colm A. Moran

**Affiliations:** a Biosafe – Biological Safety Solutions Ltd, Microkatu 1M, Kuopio, Finland; b Alltech European Headquarters, Sarney, Summerhill Road, Dunboyne, Co. Meath, Ireland; c Regulatory Affairs Department, Alltech SARL, Vire, France

**Keywords:** PFGE, Autolytic lactic acid bacteria, Bacterial typing method, *Lacticaseibacillus rhamnosus* strain typing

## Abstract

Species of lactic acid bacteria, due to their versatile metabolism, are commonly used in food and feed products, both as technological starters and as health- and welfare-promoting agents. Correct strain identification in microbe-containing products is vital, and the Pulsed-Field Gel Electrophoresis (PFGE) typing method is considered the ‘gold standard’ for this purpose. This typing technique is widely used in molecular epidemiology, especially for the early detection of emerging isolates with food-safety implications, for outbreak surveillance, and for infection control. The autolytic behavior that we encountered when typing *Lacticaseibacillus rhamnosus* strains using the PFGE technique led us to modify the current method used for typing lactic acid bacteria. This study describes a PFGE method for the molecular typing of autolytic members of the lactic acid bacteria.

• An efficient method for overcoming DNA degradation during PFGE analysis for typing *Lacticaseibacillus rhamnosus* strains is described.
• The method described herein could be considered for typing autolytic lactic acid bacteria.

## Graphical abstract

**Figure:**
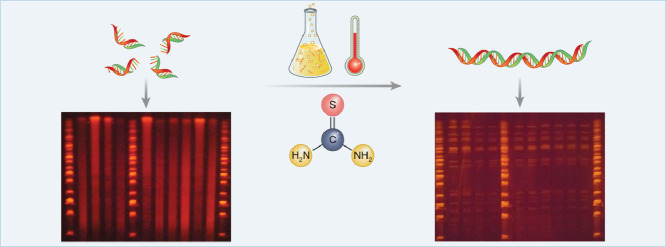

Specifications Table

**Table:** 

Subject Area:	Biochemistry, Genetics and Molecular Biology
More specific subject area:	Bacterial typing
Method name:	Pulsed-Field Gel Electrophoresis (PFGE) method for typing autolytic *Lacticaseibacillus rhamnosus* strains
Name and reference of original method:	“Animal feeding stuffs: Methods of analysis - PFGE typing of *Lactobacilli, Pediococci, Enterococci* and *Bacilli* in animal feeds” (NEN-EN 17697:2021, https://www.nen.nl/en/nen-en-17697-2021-ontw-en-286252, under development)
Resource availability:	Not applicable

## Background information

The recent reclassification of the genus *Lactobacillus* has led to the creation of 31 novel genera, subclassified into over 300 species and 55 subspecies reported in the List of Prokaryotic names with Standing in Nomenclature, “LPSN” (May 2022, www.bacterio.net). Different molecular typing tools have been used for strain identification [1]. The DNA fingerprinting by Pulsed-Field Gel Electrophoresis (PFGE) is considered to be the “gold standard” for typing of microorganisms due to the adequate consistency within a single assay, a high index of discrimination (>95), and ultimately to provide results for strain typeability [2]. The method is based on the use of restriction endonucleases that recognize 6–8-base-pair sequences, allowing the generation and electrophoretic comparison of restriction fragments larger than 50 Kb in pulsed electric fields [3]. This standard method is commonly used for the characterization of microbial feed additives due to its applicability to many genera [4]. Accordingly, PFGE has been successfully used for discriminating between different strains of lactic acid bacteria (LAB), including strains of *Lacticaseibacillus rhamnosus* [5,6,7]. However, an occasional inability to type microbial strains by PFGE due to DNA degradation (resulting in smeared and diffuse PFGE patterns) has been observed [2,8]. The major reported cause of smeared lines in PFGE gels is Tris-dependent, double-strand cleavage [9] and the addition of thiourea in the gel buffer as an efficient and reactive Tris-radical scavenger [10]. Here, we report a novel PFGE method that overcomes this problem and is able to type a non-PFGE-typeable *L. rhamnosus* strain.

## Method description

The procedure was based on a ring-tested European technical specification, “Animal feeding stuffs: Methods of analysis - PFGE typing of *Lactobacilli, Pediococci, Enterococci* and *Bacilli* in animal feeds” (NEN-EN 17697:2021,under development), with minor modifications. Tris-free buffers, a heat-treated bacterial suspension, and the inclusion of thiourea in the running buffer were used in the analysis to prevent DNA degradation due to the autolytic properties of *L. rhamnosus* strains under standard test conditions [8]. The outcomes from the non-modified and modified procedures are included in the Method Validation section.

## Reagents and equipment

de Man, Rogosa and Sharpe (MRS) broth (Neogen)

de Man, Rogosa and Sharpe (MRS) agar (Neogen)

100 mM EDTA-buffer (Sigma-Aldrich)

Lysozyme (Sigma-Aldrich)

Mutanolysine (Sigma-Aldrich)

Proteinase K (Macherey-Nagel)

Low-melting-point agarose (Thermo Scientific)

TBE Buffer (Invitrogen)

Boric acid (G-Biosciences)

EDTA/EDTA Buffer (Sigma-Aldrich)

Phenylmethylsulfonyl fluoride (Sigma-Aldrich)

N-lauroylsarcosine sodium (Sigma-Aldrich)

CutSmart restriction buffer (New England Biolabs)

AscI (New England Biolabs)

Ethidium bromide (Amresco)

Thiourea (Sigma-Aldrich)

Spectrophotometer (Hach) pH meter (Hach)

Tabletop centrifuge (Eppendorf)

Anaerobic rectangular jar (Mitsubishi)

Anaerobic gas generator (Oxoid)

Pulsed-field gel electrophoresis apparatus (CHEF-DR-III, Bio-Rad)

PhotoDoc-lt 65 imaging system with LM-26 Transilluminator (Ultra-Violet Products)

## Microbial source, culturing and preparation for the PFGE analysis

Three industrial batches containing a lyophilized *Lacticaseibacillus rhamnosus* IMI 507023 strain and a pure *Lacticaseibacillus rhamnosus* IMI 507023 lyophilized culture, obtained from Alltech and the Centre for Agriculture and Bioscience International (CABI) Culture Collection, respectively, were used for comparison purposes, whereas the strain *Lacticaseibacillus rhamnosus* DSM 20021 was used as a reference strain.

The subsamples from three industrial *L. rhamnosus* IMI 507023 batches (BO542740, BO543269, and BO543270), the pure *L. rhamnosus* IMI 507023 strain from CABI Culture Collection, and the reference strain *L. rhamnosus* DSM 20021 (type strain) were inoculated separately in MRS broth and incubated at 30°C under anaerobic conditions. After 24 h, the broth culture was subcultured on MRS agar and incubated under the same conditions for 48 h.

Colonies were transferred from MRS agar into 5 mL of ice-cold 100 mM EDTA buffer. The turbidity of the suspension (A_625_) was adjusted to 1.3 ±0.2. The suspension was heat-treated at 75°C for 10 min. An aliquot of 3.5 mL was transferred into a new 10 mL falcon tube and centrifuged at 1800 rcf for 5 min. The supernatant was discarded, and the cells were washed with 10 mL of 100 mM EDTA buffer. The cells were resuspended in 600 µL of 100 mM EDTA buffer.

### Preparation of sample agar plugs and lysis of the cells

An aliquot of 100 µL of lysozyme stock solution (150 mg/mL in 100 mM EDTA buffer) and 12.5 µL of mutanolysine (1000 U/mL in 100 mM EDTA buffer) were added to the cell suspensions, and the mixtures were incubated at 50°C for 15 min. Subsequently, 20 µL of proteinase K stock solution (20 mg/mL in 100 mM EDTA buffer) was added.

Aliquots of 300 µL of the lysed cell suspensions were mixed with equal volumes of 2% (w/v) low-melting-point agarose in 0.5 x TBE buffer (0.9 M boric acid, 0.01 M EDTA, pH 8.4) and immediately transferred into a pre-cooled plug mold, avoiding bubble formation in the wells. The plug molds were refrigerated at 4 ± 1°C for 20 min. The plugs were immersed in 5 mL of sarcosyl solution (1%, w/v, N-lauroylsarcosine sodium in 100 mM EDTA buffer) in 50 mL Falcon tubes, and 25 µL of proteinase K stock solution (20 mg/mL) was added. The original method uses 100 mM Tris-HCl instead of 100 mM EDTA buffer. The tubes were closed with screen plug caps, and the plugs were incubated at 55°C for 2 h. The plugs were washed twice with 5 mL of sterile water containing phenylmethylsulfonyl fluoride (0.175 mg/mL) and six times with 5 mL of 100 mM EDTA buffer (each wash was 15 min at room temperature). The plugs were stored in 2 mL of 100 mM EDTA buffer at 4°C.

### Digestion of chromosomal DNA

Each plug was immersed in 1000 µL of CutSmart restriction buffer for 2×30 min at room temperature. After equilibration, the plugs were immersed in 300 µL of restriction buffer containing 100 units of AscI restriction enzyme, and incubated for 1 h at 37°C. Subsequently, the restriction enzyme solution was removed, and the plugs were immersed in 500 µL of 0.5 x TBE.

### Preparation of gels and PFGE

The sectioned agarose blocks were loaded into the wells of a pulsed-field agarose gel (Bio-Rad Certified Megabase agarose, 1.0% w/v, prepared in 0.5 x TBE buffer) and subjected to transverse alternating field electrophoresis using 0.5 x TBE buffer containing thiourea (0.4 mM) and using the following parameters: 5 V/cm; switch time, 3.5–25 s for 16 h, followed by 1–5 s for 8 h at 14°C. MidRange PFG Size Marker 15–291 kb (New England Biolabs) was included for each run.

### Visualization of the gel

The gel was stained in a 0.5 x TBE solution containing 0.5 µg/mL ethidium bromide for 15 min and destained in 0.5 x TBE for 60 min. The gel was photographed with the PhotoDoc-lt 65 imaging system with the LM-26 Transilluminator (Ultra-Violet Products).

### Analysis of the results

The photographs were analyzed using the Bionumerics 8.0 software with preprocessing, lane detection, normalization of the image, and band detection. The band assignment on obtained PFGE images was firstly performed via automatic detection of bands, followed by manual adjustment, due to different intensity variations from gel to gel, which could cause errors if only automatic band assignment is used [11]. Bands smaller than 15 kb were rejected. The Dice coefficient was calculated, and the unweighted pair group method with arithmetic averages (UPGMA) was used for cluster analysis [12,13]. The tolerance and optimization of the band positional difference were 1.0% and 0.5%, respectively. A cut-off of 90% similarity of the Dice coefficient was used to determine identical PFGE types [13]. A PFGE dendrogram of the analyzed isolates/strains was constructed using the aforementioned similarity coefficient.

## Method validation

**Fig. 1:**
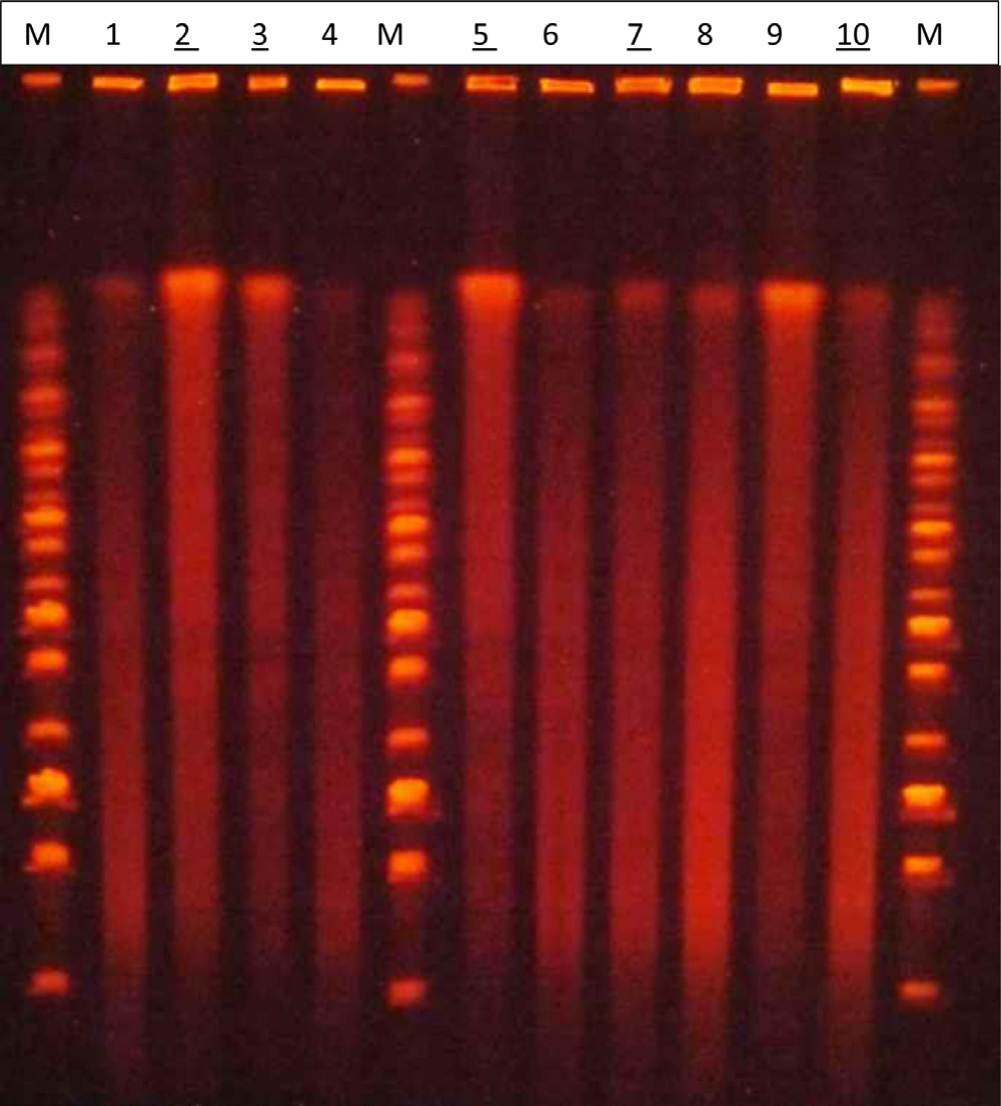

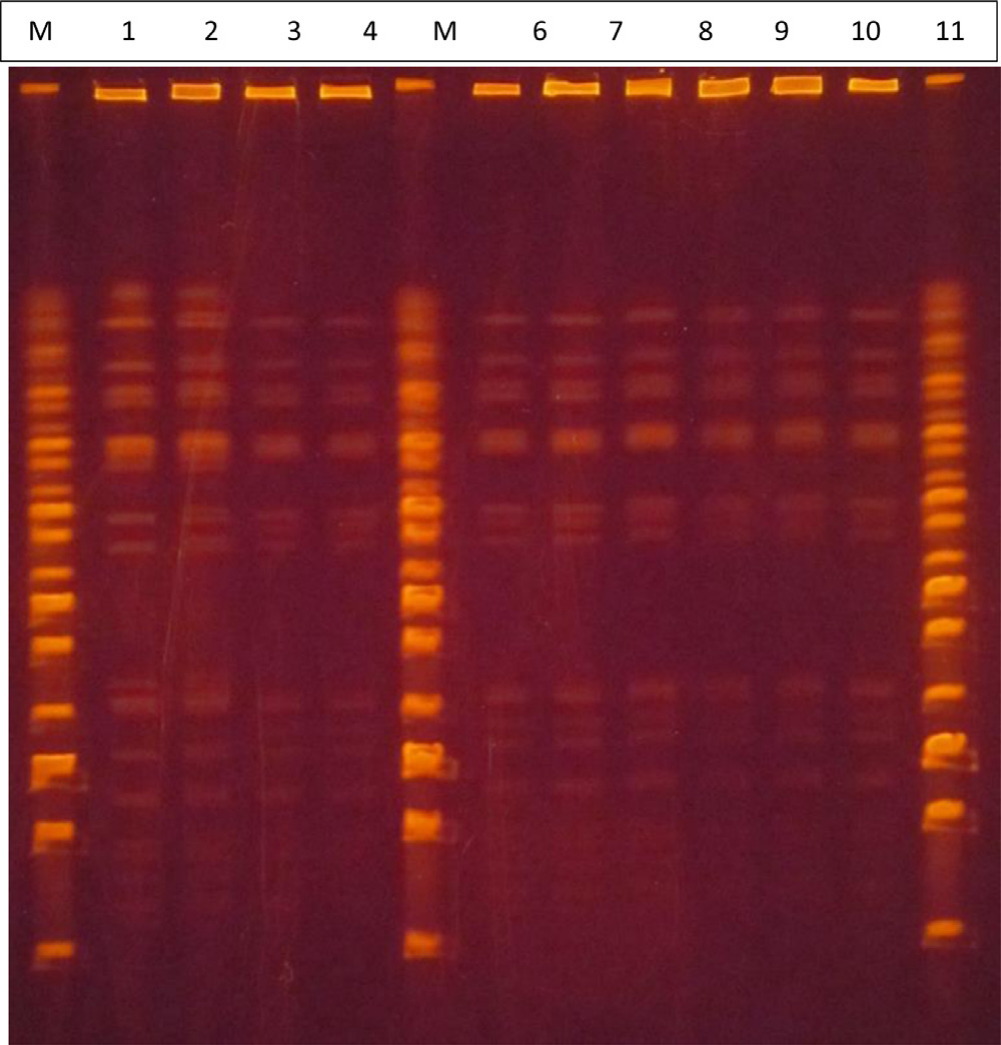
a. Comparison of the PFGE patterns of DNA, extracted and fragmented, from L. rhamnosus strains and isolates. 1-2. L. rhamnosus DSM 20021; 3-4. L. rhamnosus IMI 507023; 5-6. L. rhamnosus isolate from batch BO542740; 7-8. L. rhamnosus isolate from batch BO543; 9-10 L. rhamnosus isolate from batch BO543270; M, Midrange PFG Marker (New England Biolabs); 15 kb; 33.5 kb; 48.5 kb; 63.5 kb; 82 kb; 97 kb; 112 kb; 130.5 kb; 145.5 kb; 160.5 kb; 179 kb; 194 kb; 209 kb; 227.5 kb; 242.5 kb; 257.5 kb; 276 kb; 291 kb. b. Comparison of the PFGE patterns of DNA, extracted and fragmented, from *L. rhamnosus* strains and isolates. **1-2.***L. rhamnosus* DSM 20021; **3-4.***L. rhamnosus* IMI 507023; **5-6.***L. rhamnosus* isolate from batch BO542740; **7-8.***L. rhamnosus* isolate from batch BO543269; **9-10.***L. rhamnosus* isolate from batch BO543270; **M,** Midrange PFG Marker (New England Biolabs); 15 kb; 33.5 kb; 48.5 kb; 63.5 kb; 82 kb; 97 kb; 112 kb; 130.5 kb; 145.5 kb; 160.5 kb; 179 kb; 194 kb; 209 kb; 227.5 kb; 242.5 kb; 257.5 kb; 276 kb; 291 kb.

The outcome from the non-modified PFGE method for typing lactic acid bacteria is shown in Fig. 1a. Smeared lines could be observed for all the analyzed samples, suggesting that the microbial DNA from *L. rhamnosus* colonies was degraded.

For this purpose, the above-described modified method was used for typing *L. rhamnosus* colonies, and the original ethidium-bromide-stained PFGE gel is shown in Fig. 1b.

**Fig. 2:**
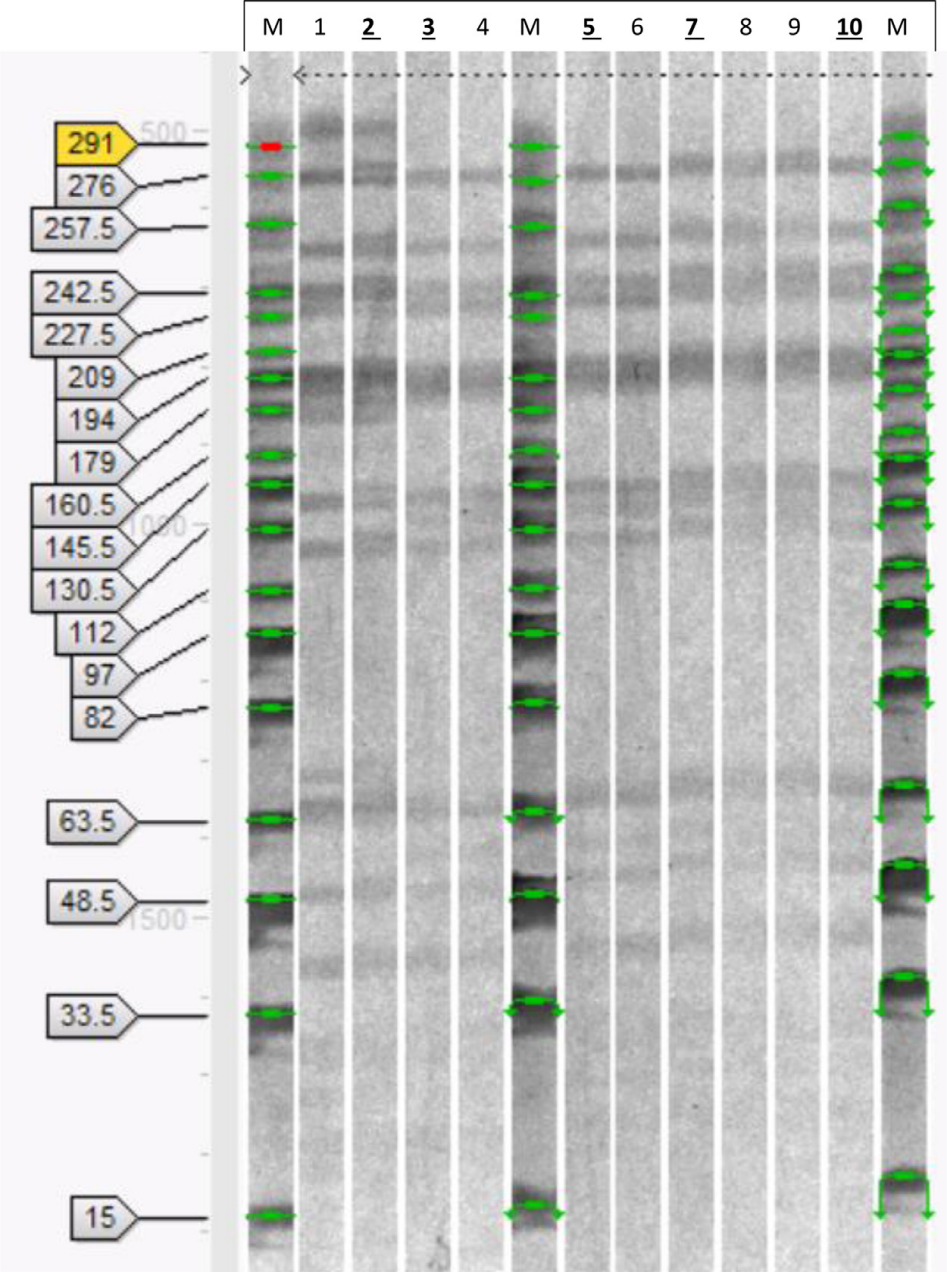

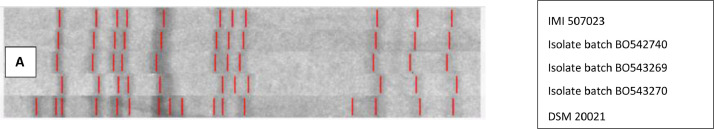
a. Comparison of the PFGE patterns of DNA, extracted and fragmented, from the *L. rhamnosus* isolates/strains. To make the comparison easier, the relevant lanes from the original image (Fig. 2) were placed here next to each other. **1-2***L. rhamnosus* DSM 20021; **3-4***L. rhamnosus* IMI 507023; **5-6***L. rhamnosus* isolate from batch BO542740; **7-8***L. rhamnosus* isolate from batch BO543269; **9-10***L. rhamnosus* isolate from batch BO543270; **M**, Midrange PFG Marker (New England Biolabs); 15 kb; 33.5 kb; 48.5 kb; 63.5 kb; 82 kb; 97 kb; 112 kb; 130.5 kb; 145.5 kb; 160.5 kb; 179 kb; 194 kb; 209 kb; 227.5 kb; 242.5 kb; 257.5 kb; 276 kb; 291 kb. The lanes used in the analysis presented in Fig. 4 are in bold and underlined above the image. b. Normalized PFGE gel image highlighting the DNA fragments (A) of the isolates/strains indicated at the right-hand side of the image. Two lanes at the bottom (DSM 20021 and isolate batch BO543270) are slightly shifted due to the normalization of the gel according to the molecular standards.

A normalized, grayscale PFGE gel image with the PFGE size marker weights is presented in Fig. 2a, whereas a comparison for the best of two duplicate sample lanes for each strain is shown in Fig. 2b.

The DNA from *L. rhamnosus* IMI 507023 and the isolates from the *L. rhamnosus* IMI 507023 batches produced eleven distinguishable bands ranging from approximately 40 to 255 kb. The coefficient of the similarity between IMI 507023 and the three isolates was 1.0 for each pairwise comparison. The type strain *L. rhamnosus* DSM 20021 produced a visually distinct pattern with sixteen distinguishable bands approximately ranging from 40 to 300 kb. The coefficients of the similarity between DSM 20021 and IMI 507023 and the three isolates varied between 0.74 and 0.82 for each pairwise comparison.

The similarity of the isolates was analyzed using the Dice coefficient. A coefficient of 1.0 represents 100% similarity for the detected bands. The coefficient of the similarity between IMI 507023 and the three isolates was 1.0 for each pairwise comparison (Table 1). The coefficients of the similarity between DSM 20021 and IMI 507023 and the three isolates varied between 0.74 and 0.82 for each pairwise comparison.

**Table 1:** Similarity coefficients for *L. rhamnosus* isolates/strains analyzed by Dice coefficients.

Isolate/Strain	DSM 20021	IMI 507023	BO542740	BO543269	BO543270
DSM 20021	1.0	0.82	0.82	0.82	0.74
IMI 507023	0.82	1.0	1.0	1.0	1.0
BO542740	0.82	1.0	1.0	1.0	1.0
BO543269	0.82	1.0	1.0	1.0	1.0
BO543270	0.74	1.0	1.0	1.0	1.0

## Conclusion

The method presented herein could be used for typing autolytic *L. rhamnosus* with the PFGE fingerprinting method.

The *L. rhamnosus* IMI 507023 strain and the isolates from the *L. rhamnosus* IMI 507023 industrial batches produced visually identical PFGE patterns, consisting of eleven distinguishable bands. The coefficient of the similarity between the isolates was 1.0, which suggests a 100% identity. It can be concluded that the isolates represent *L. rhamnosus* IMI 507023.

## Declaration of Competing Interest

The authors declare the following financial interests/personal relationships which may be considered as potential competing interests:

The authors I.N and C.A.M. are employees of Alltech which produces *Lacticaseibacillus rhamnosus* IMI 507023 evaluated in this study.

## Data Availability

Data will be made available on request.

## References

[1] Sharma A., Lee S., Park Y.S. (2020). Molecular typing tools for identifying and characterizing lactic acid bacteria: a review. Food Sci. Biotechnol..

[2] Silbert S., Boyken L., Hollis R.J., Pfaller M.A. (2003). Improving typeability of multiple bacterial species using pulsed-field gel electrophoresis and thiourea. Diagn. Microbiol. Infect. Dis..

[3] McClelland M., Jones R., Patel Y., Nelson M.l (1987). Restriction endonucleases for pulsed field mapping of bacterial genomes. Nucleic. Acids. Res..

[4] Reg (EC) No 1831/2003. European Union Register of Feed Additives. Edition 01/2022 (296). Appendixes 3e, 4 –28.01.2022.

[5] Shaaly A., Tellevik M.G., Langeland N., Høiby E.A., Jureen R. (2005). Comparison of serotyping, pulsed field gel electrophoresis and amplified fragment length polymorphism for typing of Streptococcus pneumoniae. J. Med. Microbiol..

[6] Roussel Y., Colmin C., Simonet J.M., Decaris B. (1993). Strain characterization, genome size and plasmid content in the *Lactobacillus acidophilus* group. J. Appl. Microbiol..

[7] Giraffa G., Gatti M., Rossetti L., Senini L., Neviani E. (2000). Molecular diversity within *Lactobacillus helveticus* as revealed by genotypic characterization. Appl. Environ. Microbiol..

[8] O'Reilly L.C. (2011). A method for overcoming DNA degradation during PFGE for Serratia marcescens. J. Microbiol. Methods.

[9] Evans M., Kaczmarek F.S., Stutzman-Engwall K., Dyson P. (1994). Characterization of a *Streptomyces-lividans*-type site-specific DNA modification system in the avermectin-producer *Streptomyces avermitilis* permits investigation of two novel giant linear plasmids, pSA1 and pSA2. Microbiology.

[10] Römling U., Tümmler B. (2000). Achieving 100% typeability of *Pseudomonas aeruginosa* by pulsed-field gel electrophoresis. J. Clin. Microbiol..

[11] Carrico J.A., Pinto F.R., Simas C., Nunes S., Sousa N.G, Frazão N., de Lencastre H., Almeida J.S. (2005). Assessment of band-based similarity coefficients for automatic type and subtype classification of microbial isolates analyzed by pulsed-filed gel electrophoresis. J. Clin. Microbiol..

[12] Liu X., Huang M., Zhand H., Li W., Pang Z., Lin P., Qian H. (2016). Application of pulsed-field gel electrophoresis (PFGE) in Bacillus cereus typing. Int. J. Clin. Exp. Pathol..

[13] Tynkkynen S., Satokari R., Mattila-Sandholm M.S.T., Saxelin M. (1999). Comparison of ribotyping, randomly amplified polymorphic DNA analysis, and pulsed-field gel electrophoresis in typing of *Lactobacillus rhamnosus* and *L. casei* strains. Appl. Environ. Microbiol..

